# Applications of Depth Minimization of Decision Trees Containing Hypotheses for Multiple-Value Decision Tables

**DOI:** 10.3390/e25040547

**Published:** 2023-03-23

**Authors:** Mohammad Azad, Mikhail Moshkov

**Affiliations:** 1 College of Computer and Information Sciences, Jouf University, Sakaka 72441, Saudi Arabia; 2 Computer, Electrical and Mathematical Sciences & Engineering Division and Computational Bioscience Research Center, King Abdullah University of Science and Technology, Thuwal 23955, Saudi Arabia; mikhail.moshkov@kaust.edu.sa

**Keywords:** decision tree, hypothesis, multiple value decision table, depth

## Abstract

In this research, we consider decision trees that incorporate standard queries with one feature per query as well as hypotheses consisting of all features’ values. These decision trees are used to represent knowledge and are comparable to those investigated in exact learning, in which membership queries and equivalence queries are used. As an application, we look into the issue of creating decision trees for two cases: the sorting of a sequence that contains equal elements and multiple-value decision tables which are modified from UCI Machine Learning Repository. We contrast the efficiency of several forms of optimal (considering the parameter depth) decision trees with hypotheses for the aforementioned applications. We also investigate the efficiency of decision trees built by dynamic programming and by an entropy-based greedy method. We discovered that the greedy algorithm produces very similar results compared to the results of dynamic programming algorithms. Therefore, since the dynamic programming algorithms take a long time, we may readily apply the greedy algorithms.

## 1. Background and Related Study

In traditional decision tables, each object (row) is assigned a single decision (or label). However, in some cases, such as semantic annotation of images [1], music categorization into emotions [2], functional genomics [3], text categorization [4], and so on, a sets of decisions is given to every row. If we look at the literature, we frequently find works on multi-label learning [5] and multi-instance learning [6] that discuss prediction problems for multiple-value decision tables. It is also important to include ambiguous learning [7], partial learning [8], multiple label learning [9], and semi-supervised learning [10]. Additionally, multiple-value decision tables (multi-label decision tables) can be found in research on decision trees when it is regarded as algorithms, including (i) the diagnosis of circuit faults; (ii) computational geometry and (iii) combinatorial optimization [11], etc.

In decision tables with empirical observations, we frequently encounter clusters of equal rows with, most likely, different decisions. We can preserve one row from the cluster in place of such a cluster. Then, the set of decisions associated with the rows in the cluster is used to label it [11].

In contrast to exact learning [12,13,14,15,16], which employs both membership and equivalence queries in its algorithms, conventional decision trees [17,18] solely use features which are similar to membership queries. In [19,20,21], we investigated decision trees that use hypotheses about the values of all features in addition to only features. Exact learning’s equivalence queries are comparable to hypothesis-based queries. We looked at five decision tree formations based on different combinations of features and hypotheses, as well as proper hypotheses (i.e., exact learning’s equivalents of proper equivalence queries).

In general, decision trees utilizing hypotheses can be more effective than those merely using features. As an illustration, consider the task of computing the conjunction $x_{1}\wedge \cdots \wedge x_{n}$. To solve this problem, a decision tree containing the features $x_{1},\ldots ,x_{n}$ must have a minimum depth of *n*. However, the minimum depth of a decision tree utilized to answer this problem using proper hypotheses is 1. This is needed to consider the proper hypothesis $x_{1}=1,\ldots ,x_{n}=1$. The considered conjunction is equivalent to 1 if this proper hypothesis is true. It is equivalent to 0 if not.

To find the least complexity for decision trees of five forms, we suggested dynamic programming algorithms in [19]. We also looked at the length and coverage of the decision rules obtained from the optimal decision trees. According to a number of experimental findings, decision trees with hypotheses can be less complex than conventional decision trees. Furthermore, the decision rules formed from the former trees can be shorter and more comprehensive than those formed from the latter trees. These results imply that knowledge representation could benefit from decision trees with hypotheses [19].

In this paper, we consider the problem of minimizing the depth of the decision tree. It should be noted that reducing the depth often results in reducing the length of the rules derived from the tree.

Furthermore, we look at two applications to demonstrate the use of the tools created in [19]: the task of *n* (n=3,…,5) elements sorting (elements are taken from a linearly ordered collection) where there are equal elements and the task of creating decision trees for tables that are modified and changed from the UCI ML Repository [22] to form multiple-value decision tables. We investigate the depth of five forms of optimal decision trees for each of these applications. The same parameter is investigated for five different forms of decision trees created using an entropy-based greedy method.

The comparison of decision trees containing hypotheses with traditional decision trees is the main goal of this research. The collected results from the experiments demonstrate that we can almost always find decision trees with hypotheses that outperform outcomes for traditional decision trees for both applications. Furthermore, the greedy method can be applied easily since their results are comparable to the results of dynamic programming.

The remainder of the manuscript is structured as follows: basic definitions and notation are covered in Section 2. Section 3 discusses methods to minimize the depth of the decision tree, followed by Section 4—the two applications of the created methods. Section 5 contains a short conclusion and future direction.

## 2. Preliminary Notation

The concepts of a multiple-value decision table and a decision tree for such a table are discussed in this section. Previously, such notions are described in the context of a decision table containing one decision [23] and a decision table containing many-valued decisions [11]. Finally, we describe five different forms of decision trees and one parameter of the decision tree: depth. This parameter will be used later as a comparison tool among different forms of trees.

### 2.1. Multiple Value Decision Table

**Figure 1:**
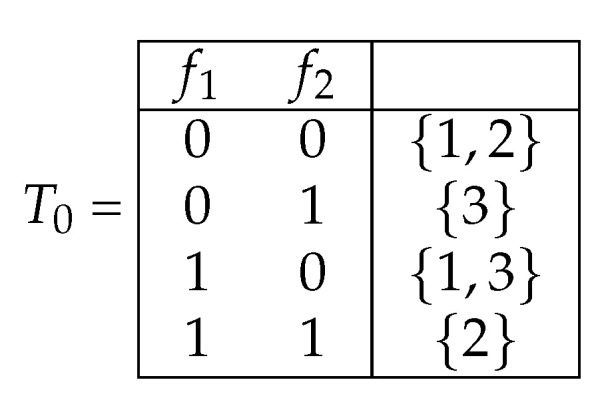
A multiple-value decision table $T_{0}$.

A multiple-value decision table is a table *T* filled with nonnegative integers. Columns in this table are tagged by features. The rows of the table are pairwise different, and they are tagged by nonempty finite sets of natural numbers that are indicated by sets of decisions. D(T) is the union of the set of decisions for all rows. Figure 1 illustrates a multiple-value decision table $T_{0}$.

We now discuss the basic notions regarding a multiple-value decision table *T*. If *T* contains no rows, it is said to be *empty*. *T* is referred to as degenerate when it is empty or if each row of *T* has a common decision (the decision which belongs to each set of decisions for all rows). Let us consider a feature $f_{i}\in \left\{f_{1},\ldots ,f_{n}\right\}$. Then $X(T,f_{i})$ is the collection of $f_{i}$’s values in the table *T*. X(T) is the collection of features that have 2 or more values i.e., $\left|X(T,f_{i})\right|\geq 2$.

**Figure 2:**
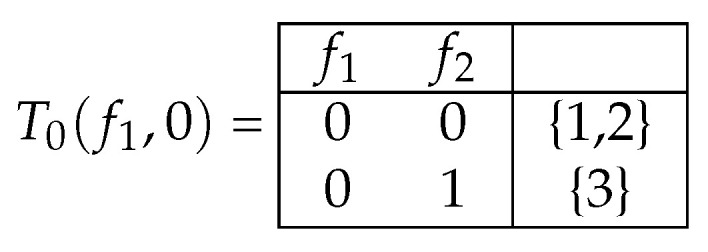
A separable subtable $T_{0}\left(f_{1},0\right)$ of $T_{0}$.

Assume *T* is a decision table that is not empty. A table made up of a few rows of *T* is a subtable of *T*. Assume that $f_{i_{1}},\ldots ,f_{i_{m}}$ are the features in *T* which must be from the set $\left\{f_{1},\ldots ,f_{n}\right\}$ and their values are $b_{1},\ldots ,b_{m}$. We consider a special subtable of the form $T\left(f_{i_{1}},b_{1}\right)\ldots \left(f_{i_{m}},b_{m}\right)$. It contains the rows that intersect with the columns $f_{i_{1}},\ldots ,f_{i_{m}}$ produces $b_{1},\ldots ,b_{m}$ values. These are referred to as separable subtables of *T*. In particular, *T* is a separable subtable of *T*. The set of separable subtables of table *T* is denoted by SEP(T). When we consider *T* analogous to an optimization problem, then separable subtables of *T* will be the analogous to subproblems of that optimization problem. Figure 2 illustrates a separable subtable $T_{0}\left(f_{1},0\right)$ of the table $T_{0}$.

### 2.2. Decision Tree

Assume *T* is a nonempty decision table containing $f_{1},\ldots ,f_{n}$ features. Decision trees for two different kinds of questions are taken into account. First, the value of a feature $f_{i}$ is inquired, where $f_{i}$ must be from $F\left(T\right)=\left\{f_{1},\ldots ,f_{n}\right\}$. The potential responses to this question are the expressions $\left\{f_{i}=b\right\}$, where *b* must be from $X(T,f_{i})$. Second, a hypothesis over the variable *T* is inquired. The hypothesis can be written as $H=\{f_{1}=b_{1},\ldots ,f_{n}=b_{n}\}$, where $b_{1}\in X\left(T,f_{1}\right),\ldots ,b_{n}\in X\left(T,f_{n}\right)$. The potential responses to this question are the hypothesis itself and counterexamples of the form $\left\{f_{1}=c_{1}\right\},c_{1}\in X\left(T,f_{1}\right)\backslash \left\{b_{1}\right\},...,\left\{f_{n}=c_{n}\right\},c_{n}\in X\left(T,f_{n}\right)\backslash \left\{b_{n}\right\}$. When the response is hypothesis then it is correct. If $\left(b_{1},\ldots ,b_{n}\right)$ is the same as the row of *T*, then *H* is denoted as a proper hypothesis for *T*.

A decision tree over *T* is a rooted tree where each terminal vertex has a number assigned to it from the set D(T)∪{0}. Every non-terminal vertex (referred to as a “functioning vertex” in this context) is tagged by either a hypothesis over *T* or a feature from the set F(T). In the case of a functioning vertex tagged by a hypothesis $H=\{f_{1}=b_{1},\ldots ,f_{n}=b_{n}\}$ over *T*, precisely one edge is tagged for each of the response $C\left(H\right)=\{H,\left\{f_{1}=c_{1}\right\},c_{1}\in X\left(T,f_{1}\right)\backslash \left\{b_{1}\right\},...,\left\{f_{n}=c_{n}\right\},c_{n}\in X\left(T,f_{n}\right)\backslash \left\{b_{n}\right\}\}$. On the other hand, if it is tagged by a feature $f_{i}$ from F(T), precisely one edge is tagged for each of the responses $C\left(f_{i}\right)=\{\left\{f_{i}=b\right\}$, $b\in X(T,f_{i})\}$. Note that just the labeled edge exits this vertex in both instances, with no additional edges doing so.

Assume *u* is a vertex on Γ, which is a decision tree over *T*. We now create an equation system over *T* that is connected to the vertex *u*, S(Γ,u). ξ represents the path from the root of Γ to *u*. Whenever ξ does not have any functioning vertices, S(Γ,u) represents the empty system. If not, S(Γ,u) is the union of equation systems linked to arcs of ξ.

The above tree is said to be a decision tree for *T* when it satisfies the following rules. For each vertex *u* of Γ, the subtable TS(Γ,u) is degenerate if and conversely, the vertex *u* is terminal. In addition, the vertex *u* receives the label 0 if TS(Γ,u) is empty and it is a terminal vertex. As opposed to that, if TS(Γ,u) is not empty when *u* is a terminal vertex, a common decision for TS(Γ,u) is labeled on the vertex *u*.

**Figure 3:**
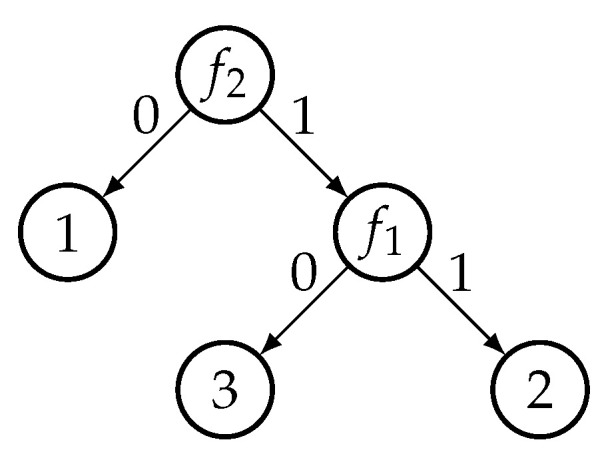
A decision tree for a multiple-value decision table $T_{0}$.

An example of a decision tree for $T_{0}$ is given in Figure 3.

### 2.3. Different Tree Forms and Corresponding Depth

In Γ, a full path is any directed path that travels from the root to a terminal vertex. The depth of the decision tree is defined as the maximum length of the tree’s full path. The depth of any considered tree is regarded as its time complexity. h(Γ) is denoted as the depth of Γ.

The following syntax will be taken into account for the lowest depth of a decision tree for *T* that:

- solely employs features from F(T) -> $h^{\left(1\right)}\left(T\right)$.
- solely employs hypotheses over *T* -> $h^{\left(2\right)}\left(T\right)$.
- employs features from F(T) as well as hypotheses over *T* -> $h^{\left(3\right)}\left(T\right)$.
- solely employs proper hypotheses over *T* -> $h^{\left(4\right)}\left(T\right)$.
- employs features from F(T) as well as proper hypotheses over *T* -> $h^{\left(5\right)}\left(T\right)$.

## 3. Procedure to Minimize Depth

In this section, first we describe how to minimize the depth of the decision tree using dynamic programming, and then we describe similar things using the greedy algorithm.

### 3.1. Extension of Dynamic Programming

#### 3.1.1. Creation of DAG

Assume *T* is a nonempty multiple-value decision table containing $f_{1},\ldots ,f_{n}$ features. We will discuss the directed acyclic graph (DAG) Δ(T) creation algorithm $A_{dag}$ (Algorithm 1). The separable subtables of table *T* make up this graph’s vertices. We deal with one vertex every iteration. We begin with the graph that has one untreated vertex, *T*, and we end when all of the graph’s vertices have been treated. We examine the untreated vertex (subtable) β at the end of each iteration; if it is degenerate, we stop splitting the vertex; otherwise, we split the vertex and carry on with the process. Previously similar procedures are described in [11,23].


**Algorithm 1:**

$A_{dag}$

**Input**: A nonempty multiple-value decision table *T* that contains $f_{1},\ldots ,f_{n}$ features.**Output**: A DAG Δ(T).
Construct a graph with only the vertex *T* that is not designated as treated.The algorithm’s work is complete once every vertex in the graph has been treated and return it as Δ(T). If not, pick a vertex (table) β that is untreated.**If** (the vertex β is degenerate) {   designate it as treated and proceed to step 2.}**else** {   Create a bundle of edges from the vertex β for each $f_{i}$ in X(β) if it is not degenerate. Let’s say $X\left(\beta ,f_{i}\right)=\left\{b_{1},\ldots ,b_{e}\right\}$. After that, create *e* arcs from β and tag them with pairs $\left(f_{i},b_{1}\right),\ldots ,\left(f_{i},b_{e}\right)$. The vertices that these edges penetrate are $\beta (f_{i},b_{1}),\ldots ,$$\beta (f_{i},b_{e})$, respectively. Add the vertices $\beta \left(f_{i},b_{1}\right),\ldots ,\beta \left(f_{i},b_{e}\right)$ to the network if they are missing. Return to step 2 and mark vertex β as treated.}


In general, $A_{dag}$’s time complexity is exponential based on the table’s size. Note that we discussed a few different classes of decision tables in the book [11]. A polynomial on the number of columns in the tables serves as a boundary for the number of separable subtables of decision tables for each of these classes. $A_{dag}$’s time complexity varies with the size of the input decision tables for each of these classes.

#### 3.1.2. Method to Minimize Depth

Assume *T* is a nonempty decision table containing $f_{1},\ldots ,f_{n}$ features. The values $h^{\left(1\right)}\left(T\right)$$,\ldots ,h^{\left(5\right)}\left(T\right)$ may be computed using the DAG Δ(T). Let’s say *t* is 1,…,5. We compute the value $h^{\left(t\right)}\left(\beta \right)$ for each vertex β of Δ(T) to determine $h^{\left(t\right)}\left(T\right)$. It is easier for us to take into account not only subtables corresponding to vertices of Δ(T), but also Λ (empty subtables of *T*), as well as $T_{r}$ (subtables that contain only a single row *r* of *T* but are not considered vertices of Δ(T)). The work of our method starts with terminal vertices of Δ(T) (vertices without exiting arcs) and these unique subtables. Then we continue moving up to the table *T* one step at a time.

Assume β is equal to $T_{r}$ for a row *r* of *T* or a terminal vertex of Δ(T). The decision tree for β is the one that has a single vertex tagged by a common decision for β, which means that $h^{\left(t\right)}\left(\beta \right)=0$. If β=Λ, the decision tree for Λ will be only one vertex labeled with 0 and in this case, $h^{\left(t\right)}\left(\beta \right)=0$.

Assume β is a nonterminal vertex of Δ(T) such that $h^{\left(t\right)}\left(\beta^{\prime }\right)$ is already known for each child $\beta^{\prime }$ of β. With the help of this information, we can determine the decision tree’s minimum depth for β. Regarding the root of this tree, we can have the following scenarios:

- A feature from F(T) is used to mark the root; the decision tree’s minimum depth is indicated by the symbol $h_{a}^{\left(t\right)}\left(\beta \right)$.
- a hypothesis over *T* is used to mark the root; the decision tree’s minimum depth is indicated by the symbol $h_{h}^{\left(t\right)}\left(\beta \right)$.
- a proper hypothesis over *T* is used to mark the root; the decision tree’s minimum depth is indicated by the symbol $h_{p}^{\left(t\right)}\left(\beta \right)$.

The set X(β) is not empty because β is nondegenerate. Here are three methods for calculating the values $h_{a}^{\left(t\right)}\left(\beta \right)$, $h_{h}^{\left(t\right)}\left(\beta \right)$, and $h_{p}^{\left(t\right)}\left(\beta \right)$, respectively.

Consider a decision tree’s root labeled with $f_{i}\in X\left(\beta \right)$ where the $\Gamma (f_{i})$ is a decision tree for β. For each $b\in X(T,f_{i})$, an edge exits the root and enters a vertex, u(b). On this edge, the equation system $\left\{f_{i}=b\right\}$ is labeled. The vertex u(b) is the root of a decision tree of form *t* for $\beta \{f_{i}=b\}$, and its depth is $h^{\left(t\right)}\left(\beta \left\{f_{i}=b\right\}\right)$. Clearly

$$h\left(\Gamma \left(f_{i}\right)\right)=1+\max\left\{h^{\left(t\right)}\left(\beta \left\{f_{i}=b\right\}\right):b\in X\left(T,f_{i}\right)\right\}.$$

Because $h^{\left(t\right)}\left(\beta \left\{f_{i}=b\right\}\right)=h^{\left(t\right)}\left(\Lambda \right)=0$ for any $b\in X\left(T,f_{i}\right)\backslash X\left(\beta ,f_{i}\right)$,

(1) $$h\left(\Gamma \left(f_{i}\right)\right)=1+\max\left\{h^{\left(t\right)}\left(\beta \left\{f_{i}=b\right\}\right):b\in X\left(\beta ,f_{i}\right)\right\}.$$

For each $b\in X(\beta ,f_{i})$, it is evident that the subtable $\beta \{f_{i}=b\}$ is β’s child in the Δ(T), therefore $h^{\left(t\right)}\left(\beta \left\{f_{i}=b\right\}\right)$’s value is known.

With the root labeled with the feature $f_{i}$ and decision trees of form *t* used for the subtables corresponding to the root’s children, it can be shown that $h(\Gamma \left(f_{i}\right))$ is the minimum depth of a decision tree for β.

The fact that there is a *b* in $X(T,f_{i})$ for each such feature and that $\beta \{f_{i}=b\}=\beta$ implies that we cannot build an optimal decision tree for β means that we should not consider these features. Thus, the depth, in this case, will be

(2) $$h_{a}^{\left(t\right)}\left(\beta \right)=\min\left\{h\left(\Gamma \left(f_{i}\right)\right):f_{i}\in X\left(\beta \right)\right\}.$$

**The procedure of $h_{a}^{\left(t\right)}\left(\beta \right)$ Computation**. Create the collection of features X(β). Calculate the value $h(\Gamma \left(f_{i}\right))$ for each feature $f_{i}\in X\left(\beta \right)$ using (1). Determine $h_{a}^{\left(t\right)}\left(\beta \right)$ with reference to (2).

Note that, this procedure’s time complexity is polynomial based on table *T*.

We consider only admissible hypothesis over *T*. Assume $H=\{f_{1}=b_{1},\ldots ,f_{n}=b_{n}\}$ is such a hypothesis. It is admissible for β and a feature $f_{i}$ in $F\left(T\right)=\left\{f_{1},\ldots ,f_{n}\right\}$ if $\beta \{f_{i}=c\}$ is not the same as β for any $c\in X\left(T,f_{i}\right)\backslash \left\{b_{i}\right\}$. It is not admissible for β and a feature $f_{i}\in F\left(T\right)$ iff $|X(\beta ,f_{i})|=1$ and $b_{i}\notin X\left(\beta ,f_{i}\right)$. Such a hypothesis is referred to as admissible for β if it is admissible for both β and any feature $f_{i}\in F\left(T\right)$.

Assume a decision tree’s root is tagged by the hypothesis $H=\{f_{1}=b_{1},\ldots ,f_{n}=b_{n}\}$ (admissible for β) where the $\Gamma (f_{i})$ is a decision tree for β. The collection of responses to the question associated with *H* is $C\left(H\right)=\{H,\left\{f_{1}=c_{1}\right\},\ldots ,\left\{f_{n}=c_{n}\right\}:c_{1}\in X\left(T,f_{1}\right)\backslash \left\{b_{1}\right\},...,c_{n}\in X\left(T,f_{n}\right)\backslash \left\{b_{n}\right\}\}$. There must be an edge that departs from Γ(H)’s root and reaches a vertex u(S) for each *S* in C(H). The equation system *S* is written on this edge. The decision tree of type *t* for βS has this u(S) as its root, and its depth is $h^{\left(t\right)}\left(\beta S\right)$. Clearly

$$h\left(\Gamma \left(H\right)\right)=1+\max\left\{h^{\left(t\right)}\left(\beta S\right):S\in C\left(H\right)\right\}.$$

Note that, $h^{\left(t\right)}\left(\beta H\right)=0$ if βH=Λ or $\beta H=T_{r}$ for any row *r* of *T*. $X\left(\beta ,f_{i}\right)\backslash \left\{b_{i}\right\}$ is equal to empty set whenever $f_{i}\in F\left(T\right)\backslash X\left(\beta \right)$ because *H* is admissible for β. It is evident that $\beta \{f_{i}=c\}=\Lambda$ for any feature $f_{i}\in X\left(\beta \right)$ and any $c\in X\left(T,f_{i}\right)\backslash \left\{b_{i}\right\}$ where $c\notin X(\beta ,f_{i})$. In this case, $h^{\left(t\right)}\left(\beta \left\{f_{i}=c\right\}\right)=0$. Hence

(3) $$h\left(\Gamma \left(H\right)\right)=1+\max\{h^{\left(t\right)}\left(\beta \left\{f_{i}=c\right\}\right):f_{i}\in X\left(\beta \right),c\in X\left(\beta ,f_{i}\right)\backslash \left\{b_{i}\right\}\}.$$

Obviously for any $f_{i}\in X\left(\beta \right)$ and any $c\in X\left(\beta ,f_{i}\right)\backslash \left\{b_{i}\right\}$, $\beta \{f_{i}=c\}$ is a child of β in Δ(T). Therefore, $h^{\left(t\right)}\left(\beta \left\{f_{i}=c\right\}\right)$ is already known.

With the root labeled by the hypothesis *H* and decision trees of form *t* used for the subtables corresponding to the root’s children, it can be shown that h(Γ(H)) is the minimum depth of a decision tree for β.

Not admissible for β hypotheses should not be taken into consideration.

**The procedure of $h_{h}^{\left(t\right)}\left(\beta \right)$ Computation**. Create the hypothesis: $H_{\beta }=\left\{f_{1}=b_{1},\ldots ,f_{n}=b_{n}\right\}$ for β. Assume that $f_{i}$ in F(T)\X(β). The sole number in the set $X(\beta ,f_{i})$ is then equal to $b_{i}$. Let’s say $f_{i}\in X\left(\beta \right)$. If so, $h^{\left(t\right)}\left(\beta \left\{f_{i}=b_{i}\right\}\right)=\max\left\{h^{\left(t\right)}\left(\beta \left\{f_{i}=c\right\}\right):c\in X\left(\beta ,f_{i}\right)\right\}$ where $b_{i}$ is the lowest number from $X(\beta ,f_{i})$. There is no doubt that $H_{\beta }$ is admissible for β. Calculate the value of $h(\Gamma \left(H_{\beta }\right))$ using (3). It is clear from a quick look at (3) that $h\left(\Gamma \left(H_{\beta }\right)\right)=h_{h}^{\left(t\right)}\left(\beta \right)$.

Note that, this procedure’s time complexity is polynomial based on table *T*.

**The procedure of $h_{p}^{\left(t\right)}\left(\beta \right)$ Computation**. Create a proper hypothesis $H_{r}$$=\{f_{1}=b_{1},\ldots ,f_{n}=b_{n}\}$ corresponding to every row $r=(b_{1},\ldots ,b_{n})$ and determine if this proper hypothesis is admissible for β. Calculate the value of $h(\Gamma \left(H_{r}\right))$ using (3). $h_{p}^{\left(t\right)}\left(\beta \right)$ is equal to the minimum of the obtained numbers.

Note that this procedure’s time complexity is polynomial based on table *T*.

Now, an algorithm $A_{t}^{h}$ (Algorithm 2) is described below that determines $h^{\left(t\right)}\left(T\right)$. It is the minimum depth of a decision tree with the provided form *t* (where, t=1,…,5) for the table *T*. As the algorithm executes, we learn $h^{\left(t\right)}\left(\beta \right)$ for each vertex β in Δ(T).

**Algorithm 2:**$A_{t}^{h}$ (computation of $h^{\left(t\right)}\left(T\right)$).**Input:** The DAG Δ(T) for a nonempty table *T*.**Output:** The value of the depth $h^{\left(t\right)}\left(T\right)$.
The algorithm will end if all vertices of the DAG Δ(T) have numbers associated to them and return the number $h^{\left(t\right)}\left(T\right)$ associated to the vertex *T*. In the absence of such a case, we select a vertex that does not have an assigned number. This vertex can be a terminal vertex of Δ(T) or a nonterminal vertex of Δ(T) where every child contains the numbers that are assigned to it.**If** (β is a terminal vertex) {   assign the value $h^{\left(t\right)}\left(\beta \right)=0$ to β and move on to step 1.}**else** {   perform the following based on the value *t*:   **If** ( t=1 ) {   calculate the value $h_{a}^{\left(1\right)}\left(\beta \right)$ and set $h^{\left(1\right)}\left(\beta \right)=h_{a}^{\left(1\right)}\left(\beta \right)$.   }   **else if** ( t=2 ) {   calculate the value $h_{h}^{\left(2\right)}\left(\beta \right)$ and set $h^{\left(2\right)}\left(\beta \right)=h_{h}^{\left(2\right)}\left(\beta \right)$.   }   **else if** ( t=3 ) {   calculate the value $h_{a}^{\left(3\right)}\left(\beta \right)$ and $h_{h}^{\left(3\right)}\left(\beta \right)$ and set $h^{\left(3\right)}\left(\beta \right)=\min\left\{h_{a}^{\left(3\right)}\left(\beta \right),h_{h}^{\left(3\right)}\left(\beta \right)\right\}$.   }   **else if** ( t=4 ) {   calculate the value $h_{p}^{\left(4\right)}\left(\beta \right)$ and set $h^{\left(4\right)}\left(\beta \right)=h_{p}^{\left(4\right)}\left(\beta \right)$.   }   **else if** ( t=5 ) {   calculate the value $h_{a}^{\left(5\right)}\left(\beta \right)$ and $h_{p}^{\left(5\right)}\left(\beta \right)$ and set $h^{\left(5\right)}\left(\beta \right)=\min\left\{h_{a}^{\left(5\right)}\left(\beta \right),h_{p}^{\left(5\right)}\left(\beta \right)\right\}$.   }}Move on to step 1.


### 3.2. Greedy Algorithms

In this section, we provide greedy techniques for building decision trees with hypotheses for multiple-value decision tables based on entropy uncertainty measure.

Assume *T* is a nonempty multiple-value decision table with *n* features $f_{1},\ldots ,f_{n}$ and β be a subtable of the table *T*. Entropy of β (denoted entML(β)) is defined as:

1. If β is empty or degenerate, then entML(β)=0.
2. Assume β is nonempty and nondegenerate. $N_{s}\left(\beta \right)$ is the number of rows in β where the set of decisions contains the decision *s* (*s* is in D(β) ). Furthermore, $p_{s}$ is the value $\frac{N_{s}\left(\beta \right)}{N(\beta )}$. Here, N(β) is the number of rows in β. Hence, $entML\left(\beta \right)=-\sum_{s\in D(T)}p_{s}\log_{2}p_{s}+\left(1-p_{s}\right)\log_{2}\left(1-p_{s}\right)$ [24].

We now define the *impurity* of a query for the table β and uncertainty measure entML. The impurity of the query based on a feature $f_{i}\in F\left(T\right)$ is equal to $I_{entML}\left(f_{i},\beta \right)=\max\left\{entML\left(\beta S\right):S\in C\left(f_{i}\right)\right\}$. The impurity of the query based on a hypothesis *H* is equal to $I_{entML}\left(H,\beta \right)=\max\left\{entML\left(\beta S\right):S\in C\left(H\right)\right\}$.

A greedy algorithm, $A_{entML}$ (Algorithm 3), is described below. It generates a decision tree, $\Gamma_{entML}^{\left(t\right)}$ of form *t* (where, ∈1,…,5) for a nonempty multiple-value decision table *T*.

**Algorithm 3:**$A_{entML}$ (construction of the tree $\Gamma_{entML}^{\left(t\right)}$).**Input:** A nonempty multiple-value decision table *T* and a number t∈{1,…,5}.**Output:** A decision tree of form *t* for *T*.
Create a tree *G* with a sole vertex that is tagged by *T*.The algorithm terminates and returns the tree *G* if none of the vertices of the tree *G* are tagged by a table *T*.Pick a vertex in the tree *G* that is tagged by the subtable β of table *T*. This vertex is named as *u*.**If** (β is degenerate) {   *u* is tagged by 0 if it is empty and with a common decision of β if it is not.}**else** {   we select an *M* query (either a feature or a hypothesis) admissible for β based on *t* according to the below rules:   **If** (t=1) {      a feature M∈F(T) is chosen that is admissible for β and has the lowest impurity, $I_{entML}\left(M,\beta \right)$.   }   **else if** (t=2) {      a hypothesis *M* over *T* is chosen that is admissible for β and has the lowest impurity, $I_{entML}\left(M,\beta \right)$.   }   **else if** (t=3) {      a feature Y∈F(T) is chosen that is admissible for β and has the lowest impurity $I_{entML}\left(Y,\beta \right)$ as well as discover a hypothesis *Z* over *T* that is admissible for β and has the lowest impurity, $I_{entML}\left(Z,\beta \right)$. We select the query *M* out of *Y* and *Z* with the minimum impurity $I_{entML}\left(M,\beta \right)$.   }   **else if** (t=4) {      a proper hypothesis *M* over *T* is chosen that is admissible for β and has the lowest impurity, $I_{entML}\left(M,\beta \right)$.   }   **else if** (t=5) {      a feature Y∈F(T) is chosen that is admissible for β and has the lowest impurity $I_{entML}\left(Y,\beta \right)$ as well as discover a proper hypothesis *Z* over *T* that is admissible for β and has the lowest impurity, $I_{entML}\left(Z,\beta \right)$. We select the query *M* out of *Y* and *Z* with the minimum impurity $I_{entML}\left(M,\beta \right)$.   }}Rather than using β, the query *M* is used to label the vertex *u*. We add a vertex u(S) as well as an arc e(S) connecting *u* and u(S) to the tree *G* for each solution S∈C(M). The answer *S* is used to label the arc e(S) and the subtable βS is used to label the vertex u(S). Move on to step 2.


The algorithm $A_{entML}$ generates a decision tree $\Gamma_{entML}^{\left(t\right)}$ of form *t* (where, t∈{1,…,5}) for the table *T*. Denote $h_{entML}^{\left(t\right)}\left(T\right)=h\left(\Gamma_{entML}^{\left(t\right)}\right)$.

## 4. Application

In this section, we look at two applications for the aforementioned algorithms. The sorting problem is the first application, and the experimental analysis of changed decision tables from the UCI Machine Learning Repository [22] is the second.

### 4.1. Sorting

The problem of sorting is usually defined in theoretical studies (see [25,26]) as sorting a series of *n* distinct elements $y_{1},\ldots ,y_{n}$ from a set that is linearly ordered. The collection {1,…,n} has only one permutation $\left(s_{1},\ldots ,s_{n}\right)$ where $y_{s_{1}}<\ldots <y_{s_{n}}$. There are only two results for any comparison of two components $y_{i}:y_{j}$: $y_{i}>y_{j}$ and $y_{i}<y_{j}$. The goal is to find a permutation $\left(s_{1},\ldots ,s_{n}\right)$ for a given sequence $y_{1},\ldots ,y_{n}$ such that $y_{s_{1}}<\ldots <y_{s_{n}}$.

Now consider the case where there are equal elements in the sequence $y_{1},\ldots ,y_{n}$. The set {1,…,n} can have more than one permutation $\left(s_{1},\ldots ,s_{n}\right)$ such that $y_{s_{1}}\leq \ldots \leq y_{s_{n}}$. There are three results for any comparison of two components $y_{i}:y_{j}$: $y_{i}<y_{j}$, $y_{i}=y_{j}$, and $y_{i}>y_{j}$. The goal is to find a permutation $\left(s_{1},\ldots ,s_{n}\right)$ for a given sequence $y_{1},\ldots ,y_{n}$ such that $y_{s_{1}}\leq \ldots \leq y_{s_{n}}$.

We can construct the multiple-value decision table $T_{sort}^{3}\left(n\right)$ for a given *n*. In this table, columns are labeled as features $y_{i}:y_{j}$, 1≤i<j≤n. Furthermore, for a series of elements $y_{1},\ldots ,y_{n}$ that can contain equal elements, rows represent all feasible tuples of values according to these elements ($T_{sort}^{3}\left(3\right)$ table is illustrated in Table 1). The collection of corresponding permutations serves as the decision set for each row. Three-valued features are taken into account, as shown by the number 3 in the syntax $T_{sort}^{3}\left(n\right)$.

Table 2 contains the lowest depth of a decision tree for $T_{sort}^{3}\left(n\right)$ when n=3,…,5 using the dynamic programming algorithm. When compared to the form 1 decision tree, the forms 2, 3, 4, and 5 decision trees produce a smaller minimum depth of decision tree.

Table 3 contains the minimum depth of a decision tree for the decision table $T_{sort}^{3}\left(n\right)$ for n=3,…,5 using the greedy algorithm. It is evident that forms 2, 3, 4, and 5 produce a smaller minimum depth (highlighted in bold format) of the decision tree compared to form 1.

Now, if we compare the results obtained by the greedy algorithms and the dynamic programming, we can see that the results are pretty close. For n=3, the results are the same. Even though, the results are better for dynamic programming when we increase *n*, it takes an exponential amount of time for the dynamic programming algorithms. Therefore, to reduce the time, we can easily apply greedy algorithms.

### 4.2. Modified Tables from UCI ML Repository

We extracted decision tables from the UCI ML Repository [22]. Then one or more features are removed. Therefore, some tables have multiple rows with the same feature values but various decisions, that are subsequently combined into a sole row identified by the collection of decisions from those rows. Before the experiment begins, some preparation steps are completed. If a feature has different values for every row, it is eliminated. The most frequent value for a feature is used to fill in any gaps if a value is missing.

In Table 4, the column ‘Name of table’ indicates the name of the new table (that we obtain after deleting features from the UCI ML Repository’s data set), the column ‘The initial table removed columns’ indicates the name of the initial table along with the indexes of features deleted from it, the column ‘Rows’ indicates the quantity of rows, the column ‘Attr’ indicates the quantity of features, the column ‘Number of decisions’ indicates the quantity of decisions in the new table, and the column ‘Distribution’ indicates a sequence of numbers @1,…,@i,…, where @i denotes the quantity of rows in *T* labeled with sets of decisions having *i* decisions.

The lowest depth of a decision tree for the above-mentioned decision tables using the dynamic programming algorithm is available in Table 5. The table’s name is in the first column, and the remaining columns have values $h^{\left(1\right)}\left(T\right),\ldots ,h^{\left(5\right)}\left(T\right)$ (lowest one is in bold format for each table). It is clear that forms 2, 3, 4, and 5 produce smaller optimum depth of a decision tree compared to the form 1 decision tree. Decision trees with the smallest depth work best for 8 decision tables when using features (form 1), 4 tables when using hypotheses (form 2), all tables when using features and hypotheses (form 3), 2 tables when using proper hypotheses (form 4), and 10 tables when using features and proper hypotheses (form 5). At the bottom, the mean and standard deviation values are calculated for each form.

The lowest depth of a decision tree for the above-mentioned decision tables using the greedy algorithm is available in Table 6. The table’s name is in the first column, and the remaining columns have values $h^{\left(1\right)}\left(T\right),\ldots ,h^{\left(5\right)}\left(T\right)$ (lowest one is in bold format for each table). It is clear that forms 2, 3, 4, and 5 produce the smaller depth of a decision tree compared to the form 1 decision tree. Decision trees with the smallest depth work best for four decision tables when using features (form 1), four tables when using hypotheses (form 2), eleven tables when using features and hypotheses (form 3), two tables when using proper hypotheses (form 4), and all tables when using features and proper hypotheses (form 5). At the bottom, the mean and standard deviation values are calculated for each form.

### 4.3. Discussion

We can compare the results in two ways. First, we can compare five different forms. It is evident from both applications that, the decision tree’s minimum depth can be optimal when forms 3 and 5 are used. Second, we can compare the results obtained by greedy algorithms and those obtained by dynamic programming. Clearly, we can see that the results are pretty close for the tables “CARS1”, “NURSERY1”, “ZOO1”, “ZOO2”, and “ZOO3”. For other tables, the results are better with dynamic programming. However, it takes an exponential amount of time for the dynamic programming algorithms; therefore, to reduce the time, we can easily apply the greedy algorithms.

## 5. Conclusions

Modified decision trees are examined in this article that employs both queries based on a hypothesis about the values of all features as well as queries based on one feature per query. To reduce the depth of these decision trees, we created dynamic programming techniques and greedy algorithms, and we considered two applications. The first application is concerned with the sorting problem, whereas the second problem is concerned with the modified decision tables in the form of multiple-value decision tables from the UCI Machine Learning repository. We can see from the experimental results that the minimum depth using features and hypotheses is optimal in both applications. Furthermore, dynamic programming produces the optimal output, which is not far from the results of the greedy algorithms using entropy uncertainty measure.

In the future, we are planning to deploy more greedy methods to investigate the suitability of the greedy uncertainty measures, which can be closest to the optimal dynamic programming results. Nevertheless, the minimum number of vertices will be studied in the future using the modified decision trees by both dynamic programming and greedy algorithms for these multiple-value decision tables.

## Figures and Tables

**Table 1:** Multiple value decision table, $T_{sort}^{3}\left(3\right)$.

$y_{1}:y_{2}$	$y_{1}:y_{3}$	$y_{2}:y_{3}$	
<	<	<	{123}
<	<	=	{123, 132}
<	<	>	{132}
<	=	>	{132, 312}
<	>	>	{312}
=	<	<	{123, 213}
=	=	=	{123, 132, 213, 231, 312, 321}
=	>	>	{312, 321}
>	<	<	{213}
>	=	<	{213, 231}
>	>	<	{231}
>	>	=	{231, 321}
>	>	>	{321}

**Table 2:** Lowest depth of decision trees for decision table $T_{sort}^{3}\left(n\right)$, n=3,…,5 using DP.

	Minimum Depth				
n	$h^{\left(1\right)}\left(T_{\mathrm{sort}}^{3}\left(n\right)\right)$	$h^{\left(2\right)}\left(T_{\mathrm{sort}}^{3}\left(n\right)\right)$	$h^{\left(3\right)}\left(T_{\mathrm{sort}}^{3}\left(n\right)\right)$	$h^{\left(4\right)}\left(T_{\mathrm{sort}}^{3}\left(n\right)\right)$	$h^{\left(5\right)}\left(T_{\mathrm{sort}}^{3}\left(n\right)\right)$
3	3	**2**	**2**	**2**	**2**
4	5	**4**	**4**	**4**	**4**
5	7	**6**	**6**	**6**	**6**

**Table 3:** Lowest depth of decision trees for decision table $T_{sort}^{3}\left(n\right)$, n=3,…,5 using greedy method.

	Minimum Depth				
n	$h^{\left(1\right)}\left(T_{\mathrm{sort}}^{3}\left(n\right)\right)$	$h^{\left(2\right)}\left(T_{\mathrm{sort}}^{3}\left(n\right)\right)$	$h^{\left(3\right)}\left(T_{\mathrm{sort}}^{3}\left(n\right)\right)$	$h^{\left(4\right)}\left(T_{\mathrm{sort}}^{3}\left(n\right)\right)$	$h^{\left(5\right)}\left(T_{\mathrm{sort}}^{3}\left(n\right)\right)$
3	3	**2**	**2**	**2**	**2**
4	6	**5**	**5**	**5**	**5**
5	10	**9**	**9**	**9**	**9**

**Table 4:** Characteristics of multiple-value decision tables.

Name of	The Initial Table	Rows	Attr	Number of	Distribution
Table	Removed Columns			Decisions	@1, @2, @3,…
cars1	cars	432	5	4	258, 161, 13
	1				
flags2	flags	176	22	6	168, 8
	1, 2, 3, 19				
flags1	flags	177	21	6	166, 10, 1
	1, 2, 3, 5, 15				
flags3	flags	184	23	6	178, 6
	1, 2, 3				
flags4	flags	190	25	6	188, 2
	1				
lymph1	lymphography	122	13	4	113, 9
	1, 13, 14, 15, 18				
lymph2	lymphography	136	14	4	132, 4
	13, 14, 15, 18				
nursery1	nursery	240	4	5	97, 96, 47
	1, 5, 6, 7				
nursery2	nursery	4320	7	5	2858, 1460, 2
	1				
zoo1	zoo-data	43	11	7	40, 1, 2
	2, 6, 8, 9, 13				
zoo2	zoo-data	44	12	7	40, 4
	2, 9, 13, 14				
zoo3	zoo-data	46	14	7	44, 2
	6, 13				

**Table 5:** Minimum depth of decision tree using DP.

	Minimum Depth				
T	$h^{\left(1\right)}\left(T\right)$	$h^{\left(2\right)}\left(T\right)$	$h^{\left(3\right)}\left(T\right)$	$h^{\left(4\right)}\left(T\right)$	$h^{\left(5\right)}\left(T\right)$
cars1	**2**	5	**2**	5	**2**
flags2	6	6	**5**	9	6
flags1	**5**	6	**5**	8	**5**
flags3	**5**	6	**5**	9	**5**
flags4	5	6	**4**	9	5
lymph1	5	5	**4**	5	**4**
lymph2	**5**	**5**	**5**	6	**5**
nursery1	**1**	3	**1**	3	**1**
nursery2	**4**	7	**4**	7	**4**
zoo1	**4**	**4**	**4**	**4**	**4**
zoo2	5	**4**	**4**	**4**	**4**
zoo3	**4**	**4**	**4**	5	**4**
mean	4.25	5.08	3.92	6.17	4.08
std	1.36	1.11	1.19	2.07	1.32

**Table 6:** Minimum depth of decision tree using greedy method.

	Minimum Depth				
T	$h^{\left(1\right)}\left(T\right)$	$h^{\left(2\right)}\left(T\right)$	$h^{\left(3\right)}\left(T\right)$	$h^{\left(4\right)}\left(T\right)$	$h^{\left(5\right)}\left(T\right)$
cars1	**5**	**5**	**5**	**5**	**5**
flags2	12	15	12	17	**11**
flags1	12	12	**10**	15	**10**
flags3	10	14	**9**	18	**9**
flags4	13	14	**7**	18	**7**
lymph1	12	10	**8**	10	**8**
lymph2	11	11	**10**	11	**10**
nursery1	**2**	4	**2**	4	**2**
nursery2	**7**	**7**	**7**	**7**	**7**
zoo1	5	**4**	**4**	6	**4**
zoo2	8	**5**	**5**	6	**5**
zoo3	**4**	6	**4**	6	**4**
mean	8.42	8.92	6.92	10.25	6.83
std	3.59	4.03	2.87	5.17	2.73

## Data Availability

The data is publicly accessible via http://archive.ics.uci.edu/ml accessed on 6 March 2023.
